# Monocular Vision-Based Underwater Object Detection

**DOI:** 10.3390/s17081784

**Published:** 2017-08-03

**Authors:** Zhe Chen, Zhen Zhang, Fengzhao Dai, Yang Bu, Huibin Wang

**Affiliations:** 1 College of Computer and Information, Hohai University, Nanjing 211100, Jiangsu, China; chenzhe@hhu.edu.cn (Z.C.); zz_hhuc@hhu.edu.cn (Z.Z.); 2 Key Laboratory of Trusted Cloud Computing and Big Data Analysis, Nanjing Xiaozhuang University, Nanjing 211100, Jiangsu, China; 3 Laboratory of Information Optics and Opto-Electronic Technology, Shanghai Institute of Optics and Fine Mechanics, Shanghai 201800, China; fzdai@siom.ac.cn (F.D.); buyang@siom.ac.cn (Y.B.)

**Keywords:** underwater object detection, monocular vision, region of interest, transmission estimation

## Abstract

In this paper, we propose an underwater object detection method using monocular vision sensors. In addition to commonly used visual features such as color and intensity, we investigate the potential of underwater object detection using light transmission information. The global contrast of various features is used to initially identify the region of interest (ROI), which is then filtered by the image segmentation method, producing the final underwater object detection results. We test the performance of our method with diverse underwater datasets. Samples of the datasets are acquired by a monocular camera with different qualities (such as resolution and focal length) and setups (viewing distance, viewing angle, and optical environment). It is demonstrated that our ROI detection method is necessary and can largely remove the background noise and significantly increase the accuracy of our underwater object detection method.

## 1. Introduction

The underwater environment is one of the most challenging conditions for object detection. The signal received by any sensor can be significantly absorbed and distorted by the water medium [1]. This significantly degrades the performance of object detection methods, leading to high false positive and false negative ratios. Moreover, in underwater environments, it is quite difficult to deploy and control sensors [2]. Many state-of-the-art devices and technologies are not suited to underwater environment operation.

In general, sonar and cameras are two typical sensors widely used for underwater object detection [3,4,5,6]. Sonar sensors are sensitive to geometrical structure information and can provide information of underwater scenes even in low- and zero-visibility environments. However, the data acquired by sonar can only present the difference of the distance over the scanning points. Other factors such as visual features are missed by this type of sensor. As a result, sonar-based systems are feasible for top-down tasks, such as hydrographic surveying and charting [7], shipwreck searching [8], and marine geological surveys [9]. In contrast to sonar, cameras can provide more types of visual information at high spatial and temporal resolutions. Prominent objects can be identified by the various visual features such as color, intensity [10], texture, and contours [11]. Recently developed binocular or multi-ocular underwater systems can generate non-scale depth maps [12,13,14]. Hence, in addition to these top-down tasks, underwater vision systems possess a better ability to handle down-top tasks where we have few prior knowledge of the current underwater scenes, such as marine ecology monitoring [15] and underwater entertainment [16]. However, vision-based underwater object detection methods have not yet provided satisfactory results, although, in contrast, sonar has more opportunities to succeed in underwater object detection.

The drawback of underwater vision systems lies in their instability for underwater object detection. Underwater images acquired by cameras suffer from intensity degeneration, color distortion, and haze effects [17]. In order to make the underwater images clear and distinguishable, several underwater image enhancement or restoration methods have been introduced into object detection models as a preprocessor before feature extraction [3,18]. However, if these image preprocessors do not adapt to the underwater optical environment, many new noise sources and false colors will be mistaken for the objects themselves.

The increasing demand for vision-based applications enhances the importance of camera-based object detection methods in underwater scenes. The monocular camera system may be a better option for underwater environments provided it is sufficiently robust to the underwater conditions. In order to reach this goal and improve the performance of the monocular vision system for underwater object detection, light transmission information is introduced as a novel cue to identify underwater objects in the region of interest (ROI). This transmission information is combined with the color and intensity features to detect the ROI, which is then filtered and segmented to produce the results of underwater object detection. 

To the best of our knowledge, our novel method in this study is the first investigation of the potential of transmission information for monocular vision-based underwater object detection. The global contrast of the color, intensity, and transmission information is combined to initially identify the ROI in underwater images. In the post-processing phase, the ROI maps are filtered and segmented to generate the final results for underwater object detection. Based on the results presented in this paper, the performance of the transmission information and ROI detection for underwater object detection can be demonstrated. Finally, the contribution of this work is the establishment of a novel image dataset for underwater object detection. More than 100 underwater videos have been collected from YouTube [19] and 800 frames have been selected to establish the dataset. These samples are diverse and different in camera type (focal length, aperture, etc.), imaging condition (viewing distance, viewing angle, background, water quality, etc.), and target object (man-made object, aquatic animal, etc.).

In Section 3, we introduce the global contrast-based ROI detection method. The dark channel-based method for light transmission estimation is proposed in Section 4. Then, in Section 5, the image segmentation method is performed on the ROI maps, producing the final underwater object detection results. Section 6 presents the results and performance of our ROI detection using separated and comprehensive features, and our underwater object detection method is then compared with state-of-the-art methods. The conclusions are presented in Section 7.

## 2. Related Works 

### 2.1. Underwater Object Detection

In contrast to the vast achievements of object detection in air, very few methods have been proposed to detect objects in underwater environments. According to the characteristics of the objects of interest, underwater object detection methods can be classified into two categories. One comprises several methods to detect man-made objects, and the other is used to detect natural aquatic objects.

For the man-made underwater object detection methods, any special features and priors of the interest objects would be crucial to distinguish them from the background. For example, Yu et al. demonstrated that a vision-based system performed well at underwater navigation. The authors tested a number of colors that are visually salient in underwater environments [20]. Lee et al. used an light-emitting diode (LED) ring with five large lights as a docking mark underwater. The docking position was identified and located by a camera loaded onto autonomous underwater vehicles [21]. Dudek et al. proposed a color correction model and introduced it into an underwater object detection system. In order to prevent the ill-posed problem in underwater image restoration, the correspondence between the raw images and corrected results were learned from the training data [22]. As an extension to this method, an object detection method was proposed by combining a number of low-complexity but moderately accurate color feature detectors [23]. The results achieved by these methods uncovered the key problems for man-made underwater object detection, including feature improvement and image correction. In order to improve the performance of the image features, a novel scale and rotationally invariant feature were extracted, enabling the vision system to identify the man-made landmarks [24]. Negre et al. compared the performance of the color and shape features for object detection and demonstrated that the color feature is unreliable in underwater scenes. Alternatively, Haar-like features were designed for detecting dock marks [25]. Aiming to enlarge the contrast between the objects and background, Lee et al. proposed an updated underwater image restoration method to process the raw input data. The contribution of the image preprocessing to underwater object detection was demonstrated by comparing the results before and after image preprocessing [26]. Kim et al. jointly used color correction, multiple-template-based object selection, and color-based image segmentation methods to update the conventional approach [27]. Rizzini et al. proposed a two-phase mechanism for man-made object detection. The first phase was established by a saliency detection method, whereas in the second phase, a low-pass filter was proposed to segment the saliency maps. The ROI detection was demonstrated to perform well across several datasets collected at different depths [28]. These studies have provided important insights into this research. However, unlike our work, the method proposed by Rizzini merely aimed to detect the man-made objects that have salient contour features. Hence, only shape features have been validated to identify the objects of interest. Moreover, the transmission information was not considered in the method proposed by Rizzini, but is highlighted in our proposed method.

Unlike the task of detecting carefully designed man-made objects, natural aquatic objects are more difficult to detect. They are visually similar to the water background because of light absorption and haze effects, and we have few priors of the natural objects in new scenes. We cannot select features specific to any one object, thus, more generalized features have been utilized. In order to address these issues, a multi-phase mechanism is used to underlie the detection method for natural aquatic objects. Some are based on the image preprocessor, whereas some introduce the phase of ROI detection before the final detection. There are also methods jointly using these two phases. For example, Edgington et al. extracted low-level spatial features to detect events of interest over multiple frames. In this work, the classical Itti model was used, which extracted the initial ROI. This method is efficient and can be performed in an unsupervised fashion. However, as the Itti model works on local features, it is very sensitive to image noise. As a result, the Itti model-based method may not work very well for underwater backgrounds [29]. Chuang et al. used the phase Fourier transform (PFT) to estimate the image saliency from which the textural features of fish are extracted. The experimental results demonstrated that the PFT method performed well at describing the textural features. However, the PFT saliency detection method cannot provide satisfactory results for object detection tasks, as it is only sensitive to the contours of the objects of interest [30]. Zhu et al. proposed an underwater object detection method based on the discriminative regional feature integration method. In this method, three features, including regional contrast, regional property, and regional background descriptors, are jointly used to establish a comprehensive saliency map for underwater images [31]. Li et al. proposed a region contrast-based method by using the image segmentation method as the preprocessor. The region segmentation method may benefit from the removal of noisy data points but will cause false detections in the high-intensity regions. As a result, the region segmentation-based method can detect all salient regions with high-intensity, however, most regions are not consistent with the ground-truth [32]. 

### 2.2. Comparison to Previous Work

Generally, the goal of our work is identical to that of natural aquatic object detection, i.e., to look for prominent objects without any priors. However, there are two main differences between our method and the previous studies in this area. One is the usage of transmission information to detect underwater objects by a monocular camera system. This is more efficient and feasible for underwater object detection. The other is ROI detection by the global contrast of underwater images with various features, including color, intensity, and transmission. This ROI detection phase not only guarantees accuracy during image segmentation but also provides a higher flexible structure in contrast to existing template-based methods.

### 2.3. Proposed Method

**Figure 1:**
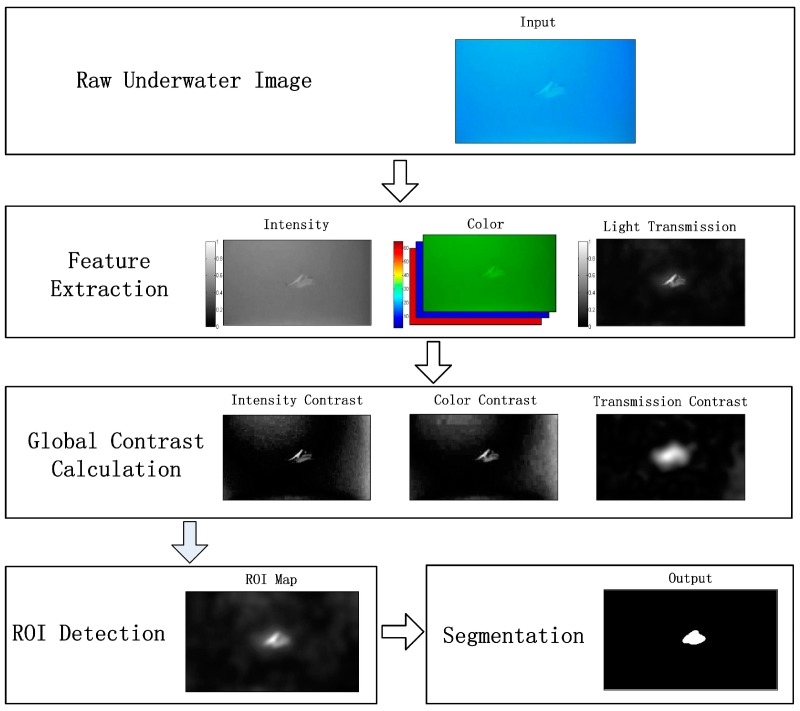
Framework of our underwater object detection method. ROI = region of interest.

The framework of our method is illustrated in Figure 1. In the first phase, various features including intensity, color, and transmission are extracted from the raw underwater images. It should be noted that the commonly used underwater image restoration or enhancement preprocessors are not introduced. This not only makes the whole system more efficient but can also prevent the influence of errors within the image processing. 

The raw underwater images have a relatively low image contrast and barely present the objects of interest in the clarity desired. In order to address this, we use the ROI to originally identify the region of the underwater objects. The global contrast of various features is calculated and combined in this phase. 

In the last phase, the extracted ROI is filtered and corrected by the image segmentation method, producing the final results of the underwater object detection. A low-scale model such as the Otsu technique is used here, demonstrating the significant contribution of our ROI detection to underwater object detection.

## 3. ROI Detection

We focus on bottom-up ROI detection using global feature contrast (Figure 1) under the assumption that an object of interest exists in an image. Motivated by the psychological realization that our visual biases are preferentially projected on the region with high contrast, our contrast calculation for ROI detection is based on the following considerations:

1. Global contrast considerations: separating natural aquatic objects from the background and highlighting the entire body of the objects.
2. Consideration of various features: detecting a reliable ROI using multiple cues including the color, intensity, and transmission features extracted from the underwater images.
3. Efficiency considerations: ROI detection should be fast, have low memory footprints, and be easy to apply in underwater scenes.

Based on these guidelines, we propose a global contrast-based method to define the values of interest. Specifically, the value of interest of a pixel is defined by its global contrast to all other pixels in a scene, i.e., the value of interest of a pixel x can formulated as

(1) $$IN_{x}=\lambda_{x}^{i}+\lambda_{x}^{c}+\lambda_{x}^{t},$$

where $IN_{x}$ is the value of interest at x; $\lambda_{x}^{i}$, $\lambda_{x}^{c}$, and $\lambda_{x}^{t}$ are the contrast metrics in intensity, color, and transmission, respectively.

The global contrast metric in intensity $\lambda_{x}^{i}$ can be formulated by the summation of the distance measurement between pixels in the gray values:

(2) $$\lambda_{x}^{i}=\sum_{\forall I_{y}\in I}D(I_{x}^{i},I_{y}^{i})=\sum_{\forall I_{y}\in I}\left\|I_{x}^{i}-I_{y}^{i}\right\|,$$

where $D(I_{x}^{i},I_{y}^{i})$ is the distance measurement between pixels x and y, calculated by the absolute difference in the gray values $I_{x}^{i}$ and $I_{y}^{i}$. 

The global color contrast metric $\lambda_{x}^{c}$ can be formulated by the summation of the Euclidean distance measurement between pixels in the L×a×b color space:

(3) $$\lambda_{x}^{c}=\sum_{\forall I_{y}\in I}D(I_{x}^{c},I_{y}^{c})=\sum_{\forall I_{y}\in I}{\left({\left(I_{x}^{c}(L)-I_{y}^{c}(L)\right)}^{2}+{\left(I_{x}^{c}(a)-I_{y}^{c}(a)\right)}^{2}+{\left(I_{x}^{c}(b)-I_{y}^{c}(b)\right)}^{2}\right)}^{1/2},$$

where $D(I_{x}^{c},I_{y}^{c})$ is the distance measurement between pixels x and y, calculated by the Euclidean distance in the L×a×b color space $\left[I_{x}^{c}(L)，I_{x}^{c}(a)，I_{x}^{c}(b)\right]$ and $\left[I_{y}^{c}(L)，I_{y}^{c}(a)，I_{y}^{c}(b)\right]$.

The global contrast metric in transmission $\lambda_{x}^{t}$ can be formulated by the summation of the distance measurement between pixels in the transmission information:

(4) $$\lambda_{x}^{t}=\sum_{\forall I_{y}\in I}D(I_{x}^{t},I_{y}^{t})=\sum_{\forall I_{y}\in I}\left\|I_{x}^{t}-I_{y}^{t}\right\|,$$

where $D(I_{x}^{t},I_{y}^{t})$ is the distance measurement between pixels x and y, calculated by the absolute difference in transmission $I_{x}^{t}$ and $I_{y}^{t}$.

In Equations (2)–(4), the intensity and color contrast originate from the underwater image data, whereas the transmission contrast in Equation (4) is based on the processing with our method.

## 4. Light Transmission Estimation

The underwater optical imaging process can be modeled as the accumulation of the formulated imaging light and hazing light [17]:

(5) $$I_{x}=B\rho_{x}\exp[-\alpha r_{x}]+B(1-\exp[-\alpha r_{x}]),$$

where $B\rho_{x}\exp[-\alpha r_{x}]$ and $B(1-\exp[-\alpha r_{x}])$ are the imaging light and hazing light, respectively, *I_x_* is the image at *x*, *B* is the ambient light, *r_x_* is the transmission at *x*, *ρ_x_* is the reflectivity at *x*, and α is the attenuation factor of the water medium.

According to the dark channel definition, the dark channel can be represented as the minimum value in any channel over the pixels in a local patch:

(6) $$I_{x}^{dark}=\underset{c\in \{r,g,b\}}{\min}(\underset{y\in \Omega_{x}}{\min}I_{y}^{c})=\underset{c\in \{r,g,b\}}{\min}(\underset{y\in \Omega_{x}}{\min}(B^{c}\rho_{y}^{c}\exp[-\alpha^{c}r_{y}]+B^{c}(1-\exp[-\alpha^{c}r_{y}]))),$$

where $I_{x}^{dark}$ is the dark channel at *x*; c is the color channel; $\Omega_{x}$ is a local patch centered at *x*; $r_{y}$ is the transmission of a pixel y in the local patch; $B^{c}$, $\rho_{y}^{c}$, and $\alpha^{c}$ are the corresponding parameters in the color channel.

Assuming that the transmission over all pixels in a local patch is homogeneous, $r_{x}=r_{y}\;\forall y\in \Omega_{x}$.

Hence; the dark channel model can be transformed as

(7) $$I_{x}^{dark}=B^{dark}\rho_{y}^{dark}\exp[-\alpha^{dark}r_{x}]+B^{dark}(1-\exp[-\alpha^{dark}r_{x}],$$

where $B^{dark}$, $\rho_{y}^{dark}$, and $\alpha^{dark}$ are the corresponding parameters in the dark channel.

According to the dark channel prior, most patches of a non-hazed image are required to include a few low-intensity pixels in at least one channel (dark channel) [33,34]. This implies that the value of the dark channel for the imaging light is low, approximating to zero:

$$B^{dark}\rho_{y}^{dark}\exp[-\alpha^{dark}r_{x}]\approx 0.$$

Hence, 

(8) $$I_{x}^{dark}=B^{dark}(1-\exp[-\alpha^{dark}r_{x}]).$$

In an underwater scene, the ambient light $B^{dark}$ can generally be assumed as homogeneous at all pixels. Therefore, the intensity of the dark channel varies exponentially with transmission:

(9) $$r_{x}=\frac{-\log(\left(B^{dark}-I_{x}^{dark}\right)/B^{dark})}{\alpha^{dark}}\;.$$

According to the dark channel model, the brightest pixel of the dark channel over all pixels in an image is a representation of the ambient light:

(10) $$\underset{z\in I}{\max}(I_{z}^{dark})=B^{dark},$$

where z is the pixel included in the underwater image. Consequently, the scene transmission estimation (Equation (9)) can be transformed as follows:

(11) $$r_{x}=\frac{-\log(\left(\underset{z\in I}{\max}(I_{z}^{dark})-I_{x}^{dark}\right)/(\underset{z\in I}{\max}(I_{z}^{dark}))}{\alpha^{dark}}\;,$$

where the attenuation factor for the dark channel light $\alpha^{dark}$ in water is commonly provided in particular tables [35]. Underwater images are commonly acquired in coastal waters listed as Type II water. There are also some images acquired in turbid inland waters, where the attenuation factor is categorized as Type IV. Based on the water type consideration, the attenuation factor of the water medium can be typically adjusted based on Ocean Type II and Lake Type IV, as follows:

(12) $$\alpha^{c}=\left\{ \begin{array}{l} 0.70\;if\;c=red\\ 0.85\;if\;c=green\\ 0.90\;if\;c=blue\; \end{array}\right.\text{ for Type II},\;\alpha^{c}=\left\{ \begin{array}{l} 0.35\;if\;c=red\\ 0.40\;if\;c=green\\ 0.50\;if\;c=blue\; \end{array}\right.\text{ for Type IV}.$$

It should be mentioned that it is difficult to exactly estimate the attenuation factor of unknown waters. Hence, our method cannot accurately estimate the depth information similar to other multi-ocular systems, whereas the relative transmission information can be obtained by our method. This transmission scale is sufficient to describe the transmission contrast between pixels. 

**Figure 2:**
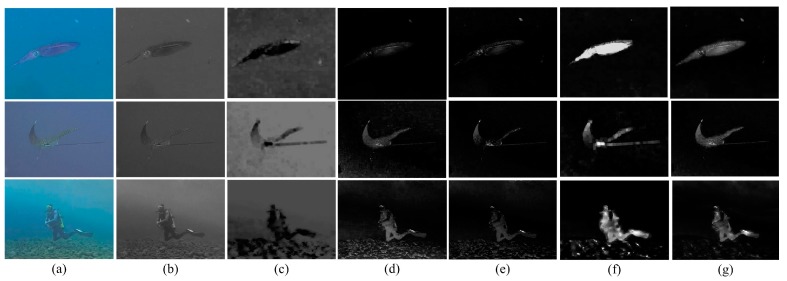
Typical samples of the region of interest (ROI) detection. (**a**) Color; (**b**) intensity; (**c**) transmission; (**d**) color global contrast; (**e**) intensity global contrast; (**f**) transmission global contrast; and (**g**) ROI detection.

The contrast calculation and ROI detection results are displayed in Figure 2. In order to fairly compare the detection performance given by different features, three typical conditions are included in Figure 2. The first row presents the conditions where the object is semitransparent and very similar to the background in hue. A typical scene where the objects are distinct from the background is presented in the second row. In the third row, significant background noise is presented in the scenes. From Figure 2, we can see that for the objects that are similar to the background (first row of Figure 2), the color and intensity contrasts between the object and background are insignificant. In this case, the transmission contrast performs well at detecting the objects, contributing most to the comprehensive ROI detection results. On the contrary, better performances are achieved by the color and intensity features if the object has a distinguishable appearance against the background (second row of Figure 2). Details of the object can be correctly depicted by the color contrast. From the third row of Figure 2, the transmission contrast, compared to the color and intensity contrasts, works more effectively at removing the background noise. It is a reasonable result as the background noise, although somewhat confused with the objects themselves, is distinguishable from the objects in the transmission scale. In general, the transmission contrast can more stably detect the ROI in all cases, indicating a good performance at detecting the entire body of the objects. The color and intensity contrasts in some cases perform better at detecting details such as textures and contours. Moreover, from Figure 2, we can see that the ROI can roughly identify the object region while much background noise is present in the edge regions and the transformation exists in the body of the detected objects. To remove these effects, image segmentation is required (Section 5).

## 5. Image Segmentation

**Figure 3:**
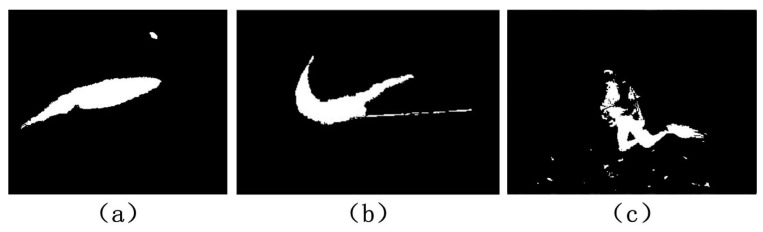
Samples of the object detection. (**a**–**c**) Results corresponding to the samples in the first, second, and third row of Figure 2, respectively.

In order to filter and correct the ROI results, here, we use the simple Otsu method to segment the ROI maps [36]. The reasons for the application of this method are two-fold. The Otsu method adapts well to the processing of the ROI maps as the object is distinguishable from the background in the gray histogram of the ROI maps. Moreover, the Otsu method is efficient and linear to the size of the maps in complexity. Samples of the segmentation results are displayed in Figure 3. From the results, it can be observed that the underwater objects are correctly detected and clearer contours are presented, closely evolving the body of objects.

## 6. Experimental Evaluation and Analysis

To demonstrate the performance of our underwater object detection method, both qualitative and quantitative evaluations are proposed in this section. We first present the global contrasts and ROI detection results in diverse underwater image data. The corresponding quantitative receiver operating characteristic (ROC) curves are also provided for the ROI. Then, the results of the object segmentation are presented and compared to other typical object detection methods performed on the underwater images, such as the Otsu [36], saliency [37], compatible color [38], contour segmentation [39], and pulse-coupled neural network (PCNN)-based methods [40]. The code for the baseline methods was downloaded from the websites provided by the authors and defaults are used for them. With the first comparison to the Otsu method, the contribution of the ROI detection to object detection can be clearly demonstrated. The Itti and compatible color-based methods have been successfully used and present exemplary performance in water, whereas the last two methods are the typical large-scale and state-of-the-art models for object segmentation in common environments. The performance of our method can be highlighted in contrast to them.

### 6.1. Dataset and Experimental Setup

In order to fairly evaluate the performance of various methods, samples in the test datasets were elaborately selected. They were all acquired by the monocular vision sensor (camera) but were diverse in quality (such as resolution and focal length) and imaging setup (viewing distance, viewing angle, and optical environments). All the tests were run using MATLAB 2013a on a Windows PC with a 2.4 GHz core and 4 GB of memory. The quantitative performance of the ROI detection is presented by the ROC [41], and the object detection after segmentation was evaluated with respect to six criteria [42]—precision (Pr), similarity (Sim), true positive rate (TPR), F-score (FS), false positive rate (FPR), and percentage of wrong classifications (PWC):

(13) $$\mathrm{Pr}=\frac{tp}{tp+ft},\;\mathrm{TPR}=\frac{tp}{tp+fn},\;\mathrm{Fs}=2\;\;\times \;\;\frac{\mathrm{Pr}\;\;\times \;\mathrm{TPR}}{\mathrm{Pr}\;\;+\;\mathrm{TPR}},\;\mathrm{Sim}=\frac{tp}{tp+fp+fn},\;\mathrm{FPR}=\frac{fp}{fp+tn},\;\mathrm{PWC}=100\times \frac{fn+fp}{tp+tn+fp+fn},$$

where tp, tn, fp, and fn denote the numbers of the true positive, true negative, false positive, and false negative, respectively. Every pixel in each testing image was used to calculate these parameters. The parameter tp was evaluated by the number of pixels that belong to the object in both the detection results and ground-truth for each image sample. The parameter tn is the number of pixels that are included in the background of both the detection results and ground-truth for each image sample. The number of background pixels in the ground-truth is used to calculate the parameter fp if they are mistaken as the object in the detection result. The parameter fn corresponds to the number of pixels that are the object in the ground truth but the background in the detection results. In each of these experiments, we kept the resolution of all inputs as the original resolution. The size of the window for the dark channel model was selected as 15 × 15. 

### 6.2. ROI Detection

**Figure 4:**
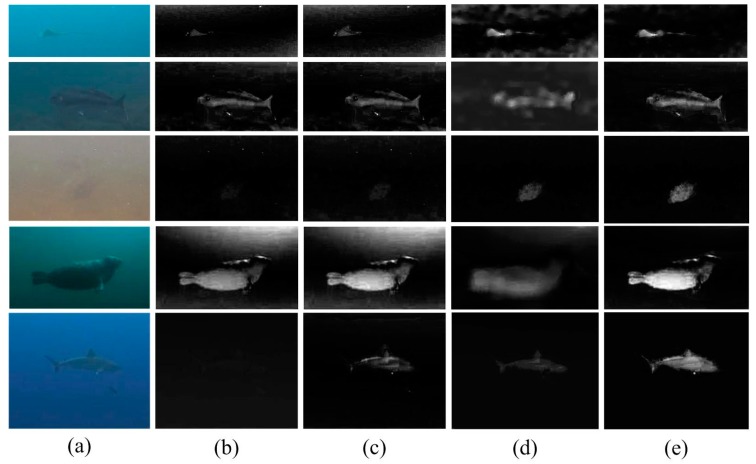
Our ROI detection results. (**a**) Underwater image; (**b**) intensity global contrast; (**c**) color global contrast; (**d**) transmission global contrast; (**e**) ROI detection.

In addition to the samples given in Section 4, more results of the ROI detection in five typical scenes are presented in Figure 4. The first and second rows display two samples acquired from the bright shallow ocean and seabed, respectively. The third row displays an image acquired in inland water. A sample acquired from the polar ocean is shown in the fourth row, while the bottom row presents a scene from the tropical ocean. Among them, the background hue and attenuation factors are distinctive. 

From Figure 4, we can see that various features provide different contrast calculation results. In some cases, the map of the transmission contrast includes many false textures, such as the results in the first and second rows. This is because some low-quality imaging sensors will cause transitions at the edges of underwater images. This effect will be exacerbated by the exponential calculation in the dark channel model. However, in other cases, the transmission contrast performs well at identifying the entire body of the objects of interest. In contrast, the color and intensity are more sensitive to the appearance of the objects. As a result, for the nearby objects, especially those that are large in size, the color and intensity contrast can correctly detect the objects, such as the results in the second and fourth rows. On the contrary, for the farther objects, the appearance of the objects is quite similar to the background because of the effects of light attenuation and scattering. In this case, color or intensity contrast cannot completely detect the objects, and several holes exist in the ROI regions, such as the results in the first, third, and fifth rows. Generally, the transmission contrast can visually detect the ROI well. In some cases, it significantly contributes to noise removal, whereas, in other cases, it benefits underwater object identification. Moreover, based on the results in Figure 4, it is surprising to find that a complementarity relationship exists between the intensity, color, and transmission features. This implies the results given by our ROI detection method are reasonable. 

**Figure 5:**
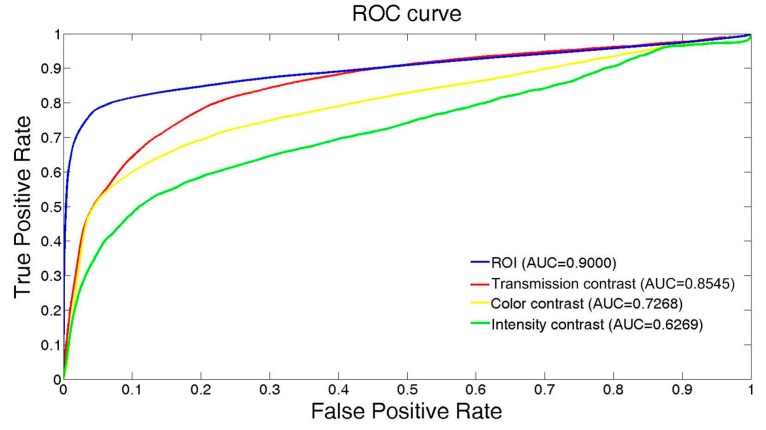
Performance evaluation of the intensity, color, and transmission contrasts, and our ROI detection method. AUC = area under the curve.

Moreover, with respect to the feature contrast and ROI detection results, the quantitative evaluations of the ROC curves on 150 diverse data samples are presented in Figure 5. The testing samples were downloaded from YouTube. The ocean water and inland lake scenes are included in the data samples. There is at least one object of interest in each image. Hence, in each sample, a meaningful evaluation can be given and the overall ROC curves are calculated by the average results. We can see from Figure 5 that our ROI detection method achieves the best result with an area under the curve (AUC) value of 0.9000. The second-best result is achieved by the transmission contrast with an AUC of 0.8545. However, relatively poor results are achieved by the color and intensity contrasts. This evaluation indicates that the ROI detection as a preprocessor will adequately benefit underwater object detection as it achieves good precision for object identification. 

In addition, from Figure 5, we can see that the transmission feature and its corresponding contrast are quite important for underwater object detection tasks. The reason underlying the degenerated performance of the intensity and color contrasts is that the light attenuation and scattering effects confuse the underwater objects with the background. As a result, the contrast between the objects and background is low and cannot clearly determine the location and the region of the underwater objects.

### 6.3. Underwater Object Detection

**Figure 6:**
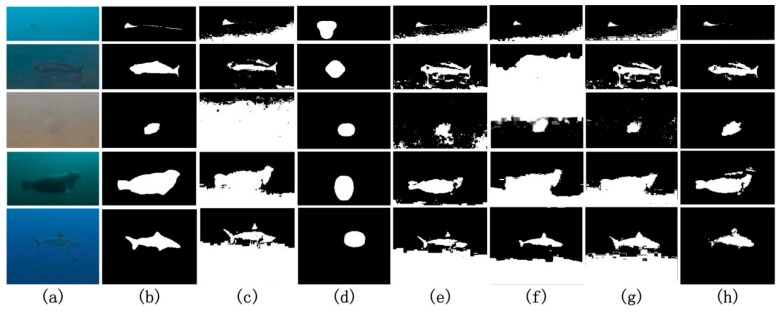
Underwater object detection. (**a**) Underwater image; (**b**) ground-truth; (**c**) Otsu; (**d**) saliency; (**e**) compatible color; (**f**) contour segmentation; (**g**) pulse-coupled neural network (PCNN); (**h**) our approach.

Figure 6 presents the experimental results of the five aforementioned methods and our method in five scenes. The first column in Figure 6 presents the original images; the second column shows the ground-truth; the third to seventh columns respectively show the results of the Otsu, saliency, compatible color, contour segmentation, and PCNN-based methods; the last column presents the results of our approach. Visually, the results produced by our approach are better than those by the other methods as our approach is the only one that can both remove the background noise and completely detect the underwater objects. A comparable robustness against the noise is given by the Itti method, which, however, mistakes a large part of the object bodies for the background. This indicates that the Itti method may be more adaptive to blob- or point-like objects under the original image size. These factors make this method vulnerable when a large object appears in the scene. From the second column, we can see that the Otsu method can barely handle the issue of inhomogeneous intensity in the underwater data, and a large part of the background with low intensity is falsely detected as the objects themselves. This result further demonstrates the importance of the ROI detection for object detection in water. The other three methods, including the compatible color, contour segmentation, and PCNN-based methods, do not perform well at removing the background noise in these underwater scenes although they succeed in many other tasks.

To further examine the quantitative performance of our method, the quantified evaluation for object detection is provided with an average of 150 samples. Table 1 summarizes the differences in the average performances of the different methods. Our method provides the best results in the six criteria and exhibits a remarkably higher performance. These evaluation results indicate that our method can adequately cater for underwater detection tasks, as a detection rate of about 50% is sufficient to identify object regions in an image [36].

From the results presented in Figure 6 and Table 1, the information fusion-based methods such as the Itti model and our model likely have more opportunities to adapt to underwater environments. They can largely remove the background noise. However, the Itti model cannot detect the entire body of the objects, especially those large in size. This issue is caused by the down-sampling process of the Itti model. For the other three image segmentation methods, such as the Otsu, contour, and PCNN methods, they are more sensitive to the local gradient of the gray value. As a result, they are susceptible to underwater backgrounds that vary in light intensity. To address this problem, the compatible color-based method tries to restore the underwater image by color compensation. However, the color compensation is unstable and will generate false information in unknown waters, which may further degenerate the object detection results. 

In contrast to all the compared methods, the complexity of our method is relative high. Our global contrast calculation requires large computing resources. However, this problem possibly can be solved by using the advanced computational hardware or optimizing the algorithm. The histogram based method for example can be used to accelerate the contrast calculation process. Due to the high time-cost color enhancement phase, the compatible color based method is the slowest one. 

## 7. Conclusions

In this paper, a novel monocular vision-based method specializing in underwater object detection is proposed. A two-phase framework is designed as an ROI detection method in the first phase and segmentation in the last phase. The framework is demonstrated to be robust in underwater environments. In addition to the commonly used color and intensity information, the transmission information of our method is introduced, which increases the correctness of underwater object detection. 

However, in some cases, especially when artificial illumination is used, the underwater optical environments are significantly polluted, and the dark channel-based model is no longer correct. In these cases, the errors in the transmission estimation may make our method unstable.

## Figures and Tables

**Table 1:** Average performance comparison of Otsu, saliency, compatible color, contour, PCNN, and our method. Precision (Pr); true positive rate (TPR); F-score (FS); similarity (Sim); false positive rate (FPR); percentage of wrong classifications (PWC).

Method	Pr	TPR	Fs	Sim	FPR	PWC
Otsu	0.3969	0.8808	0.5473	0.3767	0.2716	24.5898
Saliency	0.7847	0.3674	0.5005	0.3337	0.0201	12.2007
Compatible color	0.8068	0.6151	0.6980	0.5361	0.0318	9.4436
Contour	0.4090	0.9026	0.5629	0.3917	0.2495	22.5067
PCNN	0.3210	0.6733	0.4347	0.2777	0.2795	28.7212
Our method	0.9654	0.7260	0.8288	0.7076	0.0066	6.0863
